# Supplementary material for: From sequence to enzyme mechanism using multi-label machine learning
Source: BMC Bioinformatics. 2014 May 19;15:150. doi: 10.1186/1471-2105-15-150 (PMC4229970; doi:10.1186/1471-2105-15-150)
Supplement: Additional file 2 — Java code of ml2db. Additional file ml2db_code.tar.gz contains the Java source code to run the multi-label machine learning experiments and save the results to database. The code’s Javadoc is included. [file 1471-2105-15-150-S2.zip › additional file 2/ml2db/utils/doc/uk/ac/standrews/utils/main/RegExpUtils.html]

RegExpUtils


JavaScript is disabled on your browser.


- Overview
- Package
- Class
- Use
- Tree
- Deprecated
- Index
- Help

- Prev Class
- Next Class

- Frames
- No Frames

- All Classes

- Summary:
- Nested |
- Field |
- Constr |
- Method

- Detail:
- Field |
- Constr |
- Method


uk.ac.standrews.utils.main

## Class RegExpUtils

- java.lang.Object
- - uk.ac.standrews.utils.main.RegExpUtils

- ---

    

  ```
  public class RegExpUtils
  extends java.lang.Object
  ```

- - ### Field Summary

    Fields

    | Modifier and Type | Field and Description |
    | --- | --- |
    | `static boolean` | `m_debug` |
  - ### Constructor Summary

    Constructors

    Constructor and Description

    `RegExpUtils()`
  - ### Method Summary

    Methods

    | Modifier and Type | Method and Description |
    | --- | --- |
    | `static java.util.Vector<java.lang.String>` | `getAllMatchedSubstring(java.lang.String regExp, java.lang.String text, boolean dotall)` Returns all regular expression matches in the text (as strings) |
    | `static java.util.TreeMap<java.lang.Integer,java.lang.Integer>` | `getAllMatchedSubstringsEndpoints(java.lang.String regExp, java.lang.String text, boolean dotall)` Gets the start and end index of all regular expression matches in the text |
    | `static java.lang.String` | `getFirstMatchedSubstring(java.lang.String regExp, java.lang.String text)` Gets the first matched substring in the text (dot all mode disabled > the dot does not match the \n character) |
    | `static java.lang.String` | `getFirstMatchedSubstring(java.lang.String regExp, java.lang.String text, boolean dotall)` Gets the first matched substring in the text |
    | `static java.util.regex.Matcher` | `getMatcher(java.lang.String regExp, java.lang.String text, boolean dotall)` Get a matcher trained on the given pattern/regular expression and pointing at the text of interest. |
    | `static int` | `getNumberOfMatchedSubstrings(java.lang.String regExp, java.lang.String text, boolean dotall)` Counts the number of regular expression matches in the text |
    | `static java.lang.String[]` | `splitTextInLines(java.lang.String text)` I am splitting the String based on a regular expression which looks for carriage return (\r) or line feed (\n) or carriage return immediately followed by line feed. |
    | `static boolean` | `stringMatchesRegExp(java.lang.String string, java.lang.String regExp, boolean dotMatchesNewline)` matches a string against a regular expression (gives true if there is at least a partial match) |
    | `static java.lang.String` | `stripXmlHtmlTags(java.lang.String htmlString)` Strips all tags from xml or html code |
    | `static java.lang.String` | `substituteMatchedString(java.lang.String string, java.lang.String regExp, java.lang.String newString)` Substitute an old string with a new string if the old string matches the given regular expression |

    - ### Methods inherited from class java.lang.Object

      `equals, getClass, hashCode, notify, notifyAll, toString, wait, wait, wait`

- - ### Field Detail


    - #### m\_debug

      ```
      public static boolean m_debug
      ```
  - ### Constructor Detail


    - #### RegExpUtils

      ```
      public RegExpUtils()
      ```
  - ### Method Detail


    - #### getAllMatchedSubstring

      ```
      public static java.util.Vector<java.lang.String> getAllMatchedSubstring(java.lang.String regExp,
                                                              java.lang.String text,
                                                              boolean dotall)
      ```

      Returns all regular expression matches in the text (as strings)

      Parameters:
      :   `regExp` -
      :   `text` -
      :   `dotall` - true if the dot should match the newline character too

      Returns:


    - #### getAllMatchedSubstringsEndpoints

      ```
      public static java.util.TreeMap<java.lang.Integer,java.lang.Integer> getAllMatchedSubstringsEndpoints(java.lang.String regExp,
                                                                                            java.lang.String text,
                                                                                            boolean dotall)
      ```

      Gets the start and end index of all regular expression matches in the
      text

      Parameters:
      :   `regExp` -
      :   `text` -
      :   `dotall` - true if the dot should match the newline character too

      Returns:
      :   a map with key=start of the match, value=end of the match


    - #### getFirstMatchedSubstring

      ```
      public static java.lang.String getFirstMatchedSubstring(java.lang.String regExp,
                                              java.lang.String text)
      ```

      Gets the first matched substring in the text (dot all mode disabled > the
      dot does not match the \n character)

      Parameters:
      :   `regExp` -
      :   `text` -

      Returns:


    - #### getFirstMatchedSubstring

      ```
      public static java.lang.String getFirstMatchedSubstring(java.lang.String regExp,
                                              java.lang.String text,
                                              boolean dotall)
      ```

      Gets the first matched substring in the text

      Parameters:
      :   `regExp` -
      :   `text` -
      :   `dotall` - if true, it enables dotall mode (the dot matches also a
          newline)

      Returns:


    - #### getMatcher

      ```
      public static java.util.regex.Matcher getMatcher(java.lang.String regExp,
                                       java.lang.String text,
                                       boolean dotall)
      ```

      Get a matcher trained on the given pattern/regular expression and
      pointing at the text of interest.

      Parameters:
      :   `regExp` - a regular expression
      :   `text` - the text to be searched for regular expression matches
      :   `dotall` - true if the dot should match the newline character too

      Returns:
      :   a matcher trained on the given regular expression


    - #### getNumberOfMatchedSubstrings

      ```
      public static int getNumberOfMatchedSubstrings(java.lang.String regExp,
                                     java.lang.String text,
                                     boolean dotall)
      ```

      Counts the number of regular expression matches in the text

      Parameters:
      :   `regExp` -
      :   `text` -
      :   `dotall` - true if the dot should match the newline character too

      Returns:
      :   the number of matches


    - #### splitTextInLines

      ```
      public static java.lang.String[] splitTextInLines(java.lang.String text)
      ```

      I am splitting the String based on a regular expression which looks for
      carriage return (\r) or line feed (\n) or carriage return immediately
      followed by line feed. These takes care of all types of newlines you may
      encounter. Trailing empty strings are not included in the resulting
      array.

      Parameters:
      :   `text` -

      Returns:
      :   an array of lines


    - #### stringMatchesRegExp

      ```
      public static boolean stringMatchesRegExp(java.lang.String string,
                                java.lang.String regExp,
                                boolean dotMatchesNewline)
      ```

      matches a string against a regular expression (gives true if there is at
      least a partial match)

      Parameters:
      :   `string` -
      :   `regExp` -
      :   `dotMatchesNewline` - enables dotall mode (the dot matches also a newline)

      Returns:
      :   true if there is at least a match between the string and the
          regular expression, false otherwise


    - #### stripXmlHtmlTags

      ```
      public static java.lang.String stripXmlHtmlTags(java.lang.String htmlString)
      ```

      Strips all tags from xml or html code

      Parameters:
      :   `htmlString` -

      Returns:
      :   text stripped of tags


    - #### substituteMatchedString

      ```
      public static java.lang.String substituteMatchedString(java.lang.String string,
                                             java.lang.String regExp,
                                             java.lang.String newString)
      ```

      Substitute an old string with a new string if the old string matches the
      given regular expression

      Parameters:
      :   `string` - the old string
      :   `regExp` - a regular expression
      :   `newString` - the new string to be substituted

      Returns:
      :   1. null if the string does not match the regular expression, 2.
          the new string if the old string matches the regexp + the new
          string is non null/empty, 3. the old string if the old string
          matches the regexp but the new string is null/empty


- Overview
- Package
- Class
- Use
- Tree
- Deprecated
- Index
- Help

- Prev Class
- Next Class

- Frames
- No Frames

- All Classes

- Summary:
- Nested |
- Field |
- Constr |
- Method

- Detail:
- Field |
- Constr |
- Method
